# Lesson from Ecotoxicity: Revisiting the Microbial Lipopeptides for the Management of Emerging Diseases for Crop Protection

**DOI:** 10.3390/ijerph17041434

**Published:** 2020-02-23

**Authors:** Deepti Malviya, Pramod Kumar Sahu, Udai B. Singh, Surinder Paul, Amrita Gupta, Abhay Raj Gupta, Shailendra Singh, Manoj Kumar, Diby Paul, Jai P. Rai, Harsh V. Singh, G. P. Brahmaprakash

**Affiliations:** 1 Plant-Microbe Interaction and Rhizosphere Biology Lab, ICAR-National Bureau of Agriculturally Important Microorganisms, Maunath Bhanjan 275103, India; deeptimalviya77@gmail.com (D.M.); pramod15589@gmail.com (P.K.S.); udaiars.nbaim@gmail.com (U.B.S.); surinderpaulsandhu@gmail.com (S.P.); amritasoni90@gmail.com (A.G.); abhayrajmau@gmail.com (A.R.G.); singh.shailendra512@gmail.com (S.S.); bt.manojk@gmail.com (M.K.);; 2 Pilgram Marpeck School of Science, Technology, Engineering and Mathematics, Truett McConnel University, 100 Alumni Dr., Cleveland, GA 30528, USA; dpaul@truett.edu; 3 Department of Mycology and Plant Pathology, Institute of Agricultural Sciences, Banaras Hindu University, Varanasi 221005, India; drjaibhu@gmail.com; 4 Department of Agricultural Microbiology, University of Agricultural Sciences, GKVK, Bengaluru, Karnataka 560065, India

**Keywords:** lipopeptides, *Bacillus* spp., biosurfactant, antimicrobials, biocontrol

## Abstract

Microorganisms area treasure in terms of theproduction of various bioactive compounds which are being explored in different arenas of applied sciences. In agriculture, microbes and their bioactive compounds are being utilized in growth promotion and health promotion withnutrient fortification and its acquisition. Exhaustive explorations are unraveling the vast diversity of microbialcompounds with their potential usage in solving multiferous problems incrop production. Lipopeptides are one of such microbial compounds which havestrong antimicrobial properties against different plant pathogens. These compounds are reported to be produced by bacteria, cyanobacteria, fungi, and few other microorganisms; however, genus *Bacillus* alone produces a majority of diverse lipopeptides. Lipopeptides are low molecular weight compounds which havemultiple industrial roles apart from being usedas biosurfactants and antimicrobials. In plant protection, lipopeptides have wide prospects owing totheirpore-forming ability in pathogens, siderophore activity, biofilm inhibition, and dislodging activity, preventing colonization bypathogens, antiviral activity, etc. Microbes with lipopeptides that haveall these actions are good biocontrol agents. Exploring these antimicrobial compounds could widen the vistasof biological pest control for existing and emerging plant pathogens. The broader diversity and strong antimicrobial behavior of lipopeptides could be a boon for dealing withcomplex pathosystems and controlling diseases of greater economic importance. Understanding which and how these compounds modulate the synthesis and production of defense-related biomolecules in the plants is a key question—the answer of whichneeds in-depth investigation. The present reviewprovides a comprehensive picture of important lipopeptides produced by plant microbiome, their isolation, characterization, mechanisms of disease control, behavior against phytopathogens to understand different aspects of antagonism, and potential prospects for future explorations as antimicrobial agents. Understanding and exploring the antimicrobial lipopeptides from bacteria and fungi could also open upan entire new arena of biopesticides for effective control of devastating plant diseases.

## 1. Introduction

Crop plants are damaged every year by phytopathogens, leading to enormous economic losses to farmers across the world. Currently, themost effective available control measure for plant diseasesthroughchemical pesticideshasresulted intoxic effectsonnon-target organismsandas these compounds arenon-biodegradable in nature, this has become a matter of serious concern for contemporary environmentalists. Since chemical control is not sustainable and is almost certain to cause environmental pollution [1,2,3], different microbiological agents and biologically active molecules are beingexplored for their potential to inhibit thegrowth of phytopathogensand alleviation of other stresses [4,5,6,7,8] to crop plants. These bioactive compounds are produced by microorganisms, specificallyby the *Bacillus* genus whichis considered one of the most important bioactive compound factories [2,3,8]. Plant diseases caused by phytopathogenshave beenone of the most important and emerging categories of threats to global food security [9,10]. Microbiome associated with the plants is known to produce a structurally diverse group of compounds with hydrophilic and hydrophobic moieties and of which exhibitsbiosurfactant activity. These biosurfactants include lipopeptides, glycolipids, phospholipids, polysaccharide-protein complexes, neutral lipids, and fatty acids [11].

Due to the enormous variation in the chemical structures, lower toxicity to non-targets, biodegradability, and effectiveness to be functional under extreme environmental conditions such as high pH, extreme temperature, salinity, drought, metal stress, etc., these bio-surfactants qualifythe parameters set for asuitable green and eco-friendly alternative as compared to their synthetic counterparts for managing phytopathogens and reduce crop losses therefrom. Duetothese properties, they have gained much attension in applied sectors ranging from pharmaceutical, cosmetics, agriculture, oil recovery, and food industriestothe activities related to environmental remediation [12,13,14,15,16] Lipopeptides are defined as cyclic, low molecular weight compounds withantimicrobial potential largely produced by *Bacillus* and *Pseudomonas* spp. [17,18]. In general, the molecular weight of lipopeptides ranges from 1000–2000 Da. They are synthesized by specific gene clusters, namely nonribosomal peptides synthetase (NRPs) *via* a multi-enzyme biosynthesis pathway [19]. Surfactin, iturin, and fengycin are the three major families reported from *Bacillus* groups and are mainly composed of a hydrophilic amino acid (7–10 amino acids) linked with a hydrophobic fatty acid tail. Aneurinifactin is a group of lipopeptide reported from marine bacterium *Aneurinibacillus aneurinilyticus* isolated from the Gulf of Mannar [20]. Moreover, several lipopeptides such as iturin [21], surfactin [22], sophorolipids [23], rhamnolipids [24], trehalose lipid [25], and mannosylerythritol lipids [26] exhibited antifungal, antibacterial, or antitumor activities, signifying their utility as potent alternativesof conventional therapeutic agents and biocontrol agents for use in various biomedical and agriculturalapplications [15,24]. Surfactins consist of seven amino acids linked to one unique hydroxy fatty acid, whereas iturins consist of seven amino acids linked to one unique amino acid. The chemical composition of fengycins reveals that it consists of 10 amino acids linked to one unique hydroxy fatty acid. These lipopeptides are the most important factors contributing to their biocontrol potential in the plant growth-promoting microorganisms [17,18,22]. 

These cyclic lipopeptides produced by different microorganisms retain antiviral, antifungal, antibacterial, biofilm-forming, and plant resistance-inducing activities. Several reports indicate that these small molecular weight lipopeptides facilitate root colonization in many plants [8,9,10,15,16]. They act as potential antagonists by direct inhibition of phytopathogens through different mechanisms and/or by stimulating and strengthening plant defense machinery of defense-related networks known as induced systemic resistance (ISR) [16]. Recently, biocontrol agents of microbial origin have gained much attention for soil-borne disease control [27]. These bioagents synthesized and secreted several diffusible and volatile organic compounds in the rhizosphere and plant system and these compounds are the most important factors contributing to the biocontrol activity [28] of the producer organism. These biomoleculesare chiefly characterized for their antagonistic activity against plant pathogens of different crops [27,29,30]. There are some cyclic lipopeptides which have recently been identified as elicitors of plant defence response such as ISR [7,16]. These biomolecules play a key role and modulate various mechanisms underlying the defense responses or, more specifically, ISR, both directly and indirectly. Moreover, cyclic lipopeptides may be involved anywhere in the three-step process of ISR, *viz.* the perception of bacterial elicitor, systemic signal transduction, and, finally, defense gene expression in the host system [8,9,10]. However, the role of plant growth-promoting rhizobacteria (PGPR) in the signaling and induction of defense mechanisms isbeing well documented [31,32]. Until now, very little wasknown about mechanisms as to how the lipopeptides elicit ISR and activate different cascades of pathways after early interaction with the plant cell. Some molecules accountable for the ISR-eliciting activity may be cell-surface components [33,34,35], volatiles [36], iron-regulated metabolites [37,38,39], antibiotics compounds [40,41,42,43], and quorum-sensing signals [44]. Tran et al. [45] reported that “massetolide” produced by *Pseudomonas fluorescens* SS101 elicit ISR-reactions in tomatoeschallenged with *Phytophthora infestans.*

Similarly, members of the surfactins and fengycins families also act as elicitors of ISR by supplementing and eliciting the host resistance potential [46]. Members of the surfactin family show structural variability among them with good emulsifying and foaming properties. This is in contrast to the various investigations conducted with some PGPRs and pathogen-associated molecular patterns (PAMPs) used to decipher the events for ISR development taking place after lipopeptide inoculation [47,48,49]. However, very little information is available about the role of lipopeptides as elicitors of plant defense in controlling pathogen invasion. Although, they are known to act as chemical barriers and thus, slow down or inhibit pathogen colonization [50].

## 2. Isolation and Characterization of Lipopeptides

### 2.1. Isolation and Purification of Lipopeptides

**Figure 1:**
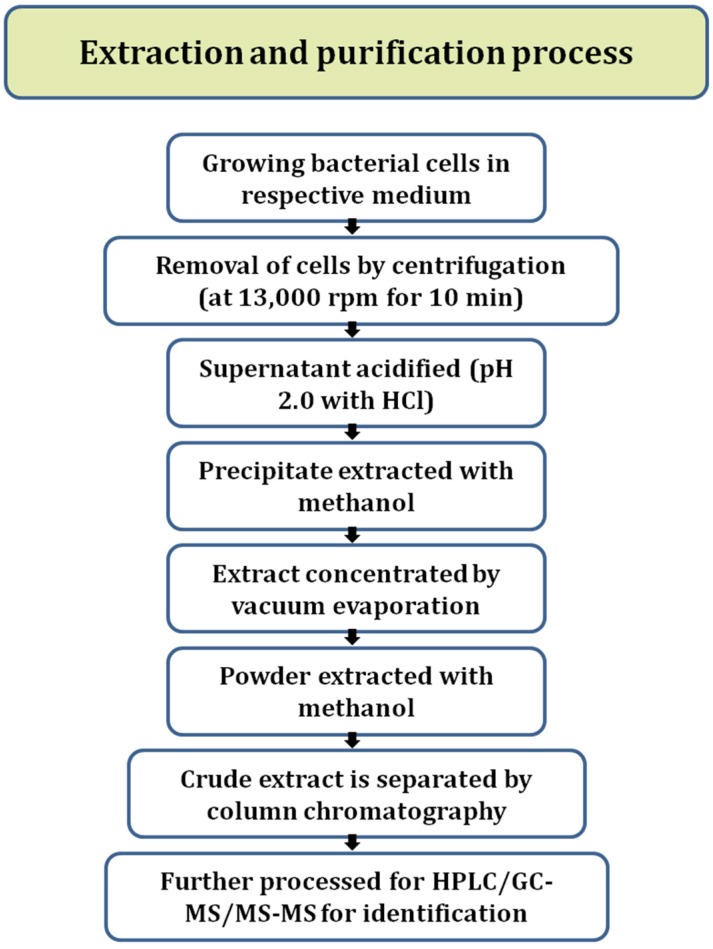
Flow chart diagram of lipopeptide extraction and the purification process.

Lipopeptides are chiefly synthesized by microorganisms belongingto bacterial genus *Bacillus* [51] by multimodular enzymes that are NRPSs. The cells are grown in their respective media and are allowed to produce lipopeptides (Figure 1). These lipopeptides are separated from cells bycentrifugation. Malfanova et al. [52] grew the bacterial cells for 60 h at 28 °C and then centrifuged at 13,000 rpm for 10 min to remove cells and obtain crude lipopeptides. After this, the supernatantwasacidified (pH 2.0) using concentrated HCl and acid precipitate wasextracted with methanol. The extract obtained wasconcentrated by vacuum evaporation. This can also be done by lyophilization. The resulting material wasdissolved in 1/50 of the initial volume of methanol or may be dissolved in PBS for further experimentations [52,53,54]. The crude extract wasthen purified by different methods of chromatography like gel filtration in Sephadex column using methanol, HPLC, etc. and the collected eluent wasused for identification using MALDI-TOF-MS, LC-MS, or MS-MS, comparing retention time and molecular masses. Different elution programs are used for the quantification of different families of lipopeptides [55]. 

### 2.2. Molecular Characterization of Antifungal Lipopeptides

Lipopeptide producing genes are well characterized and studies on specific lipopeptides can be done with great efficiency by a PCR based approach using specific primer pairs [56,57]. Some of the validated primer sets are listed in Table 1. The presence and expression of these lipopeptide producing genes can be assessed by PCR and real time PCR (qPCR) based studies, respectively. A large population can be screened quickly using these kinds of primer sets for the presence of different lipopeptides producing genes. The expression of these genes during antagonism is determined by RT-PCR. RNA is isolated under different treatment conditions. The purity and concentration of isolated RNA isolates are determined by absorbance recording at 260/280 nm or using nanodrop and are diluted to a common concentration in all the treatments. The RNA is then converted to cDNA, which is further used as a template in RT-PCR flowing standard protocols. After PCR amplification, the PCR products are loaded on to 1.2% agarose gel and the eluted products were applied for Sanger’s dideoxy sequencing and similar sequence can be obtained by using ‘BLAST’ (Basic Local Alignment Search Tool) available in NCBI. 

## 3. Different Classes of Lipopeptides 

**Figure 2:**
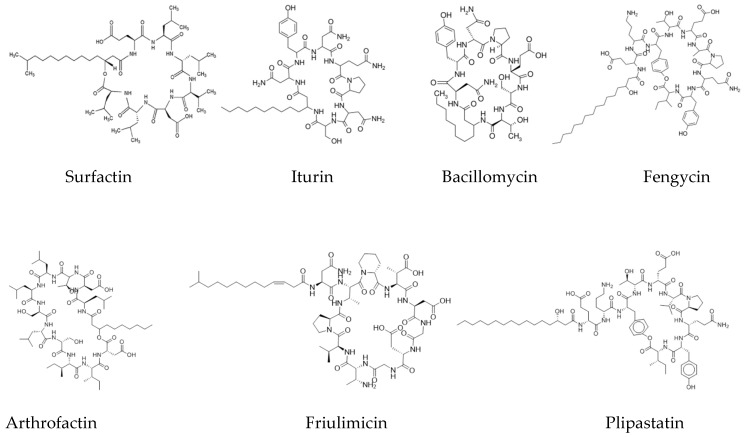

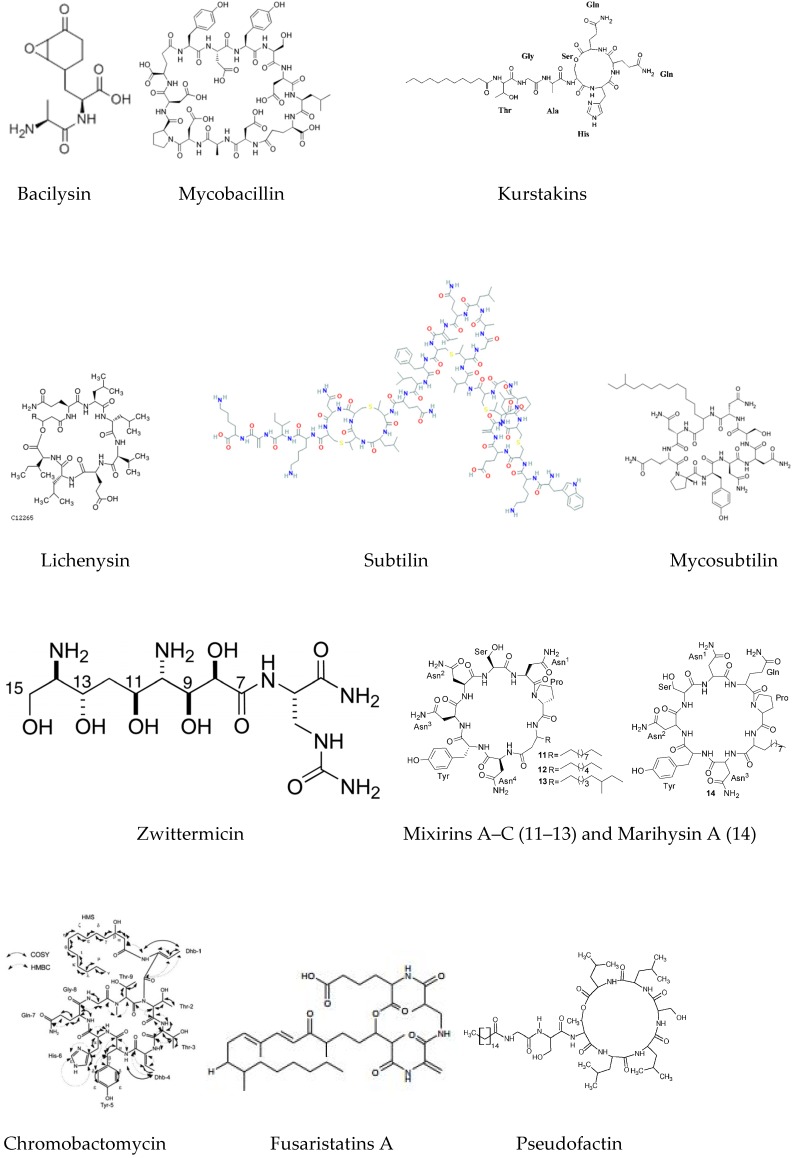

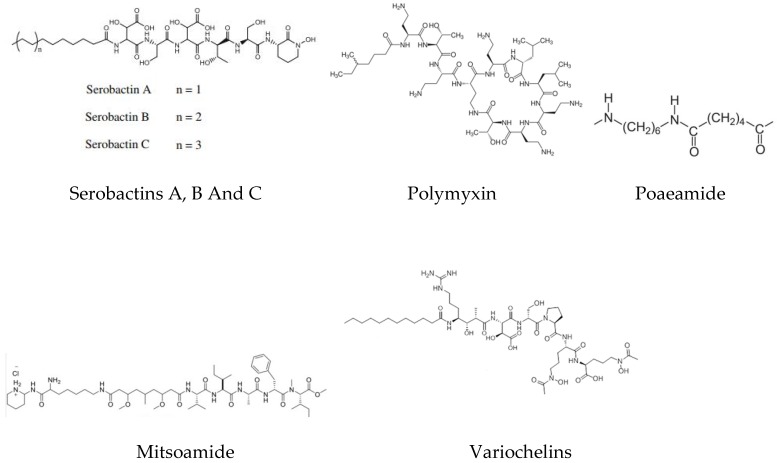
Chemical structures of some important lipopeptides produced by microbial inoculants in the natural ecosystem. Source: PubChem (https://pubchem.ncbi.nlm.nih.gov).

Lipopeptides are a group of microbial surfactants such as surfactin, lichenycin, iturin, and fengycin [70]. From previous studies, lipopeptides from the surfactin, iturin, and fengycins families are characterized in Figure 2. These are majorly produced by *Bacillus* spp., including *B. subtilis*, *B. amyloliquefaciens*, *B. licheniformis*, *B. globigii*, *B. pumilus*, *B. cereus*, *B. megatarium*, and *B. thurigiensis* [71,72,73,74]. Based on amino acid sequences, they have been broadly classified into three major groups: surfactins, iturins, and fengycins. The following are major classes of lipopeptides.

### 3.1. Iturins

The antifungal cyclic lipopeptide produced by *Bacillus* spp. is low molecular weight lipopeptide iturin. Iturin have antimicrobial potential against plant pathogens [75]. The biopesticide and fungicidal properties of iturins are realized by interacting with sterol components of the cell membrane of phytopathogenic fungus. Compounds of the iturin family are characterized by a peptide ring of seven amino acids, which shows high polymorphism resulting in varied biological and physico-chemical properties. The vast diversity of this family includes iturin A, iturin C, iturin D, iturin E, bacillomycin D, bacillomycin F, bacillomycin Lc, mojavensin A, and mycosubtilin [76]. Iturin A causes potassium ion-conducting channels in lipid bilayers as a mechanism of antagonism. Some of the members like mojavensin A, however, show cytotoxic activities [77]. 

### 3.2. Surfactins

Surfactins showed high antifungal and antibacterial activities and constitute one of the major classes of antibiotic lipopeptides produced by *Bacillus* spp. [19,78]. It is biosurfactant and important for biofilm production and its stability. Produced by *Bacillus subtilis*, it has been proven to be cidal to the bacterial phytopathogen, *Pseudomonas syringae* pv. *tomato* in *Arabidopsis* [79]. In the structure of surfactins, a cyclic lactone ring is formed which contains amphipathic cyclic lipoheptapeptide of Glu-Leu-Leu-Val-Asp-Leu-Leu (ELLVDLL) fatty acid chain and another sequence chiral to this (LLDLLDL) which is interlinked with 12-16-C β-hydroxy [80]. A change in the size and order of amino acids alsocauses variation in the lipid portion [81]. The amino acids and β-hydroxy fatty acids in a given surfactin not only vary with the producer bacterial strain, but the culture conditions also contribute to the diversity [82]. Surfactins affect the lipid bilayer of membranes and thus, are effective against both Gram-positive and Gram-negative bacteria. It is also thought to have anti-mycoplasma, antiviral, and antitumor activities and canalso suppress inflammatory responses through the inhibition of phospholipase A2 [82,83]. In biocontrol, it cansuppress phytopathogens like *Pseudomonas syringae*, *Xanthomonas axonopodis*, *Sclerotinia sclerotium, Botrytis cinerea, Colletotrichum gloeosporioides*, and also activated ISR [84,85]. 

### 3.3. Fengycins

Fengycins are a family of lipopeptides produced by members of genera *Bacillus* and *Paenibacillus*. They have antifungal activity and affect filamentous fungi [86], the most important group of phytopathogens. Structurally, fengycins are decapeptides, with14-19-C attached to a β-hydroxy fatty acid chain exhibiting strong antifungal activity [87,88]. This third family of lipopeptides is also known as plipastatin. They arealso involved in the triggering systemic response against plant pathogens. There are two classes of Fengycins—Fengycin A and Fengycin B. Both classes differ from each other by the amino acid attached at position 6. Fengycin A contains Ala at position 6, whereas Fengycin B contains Val in this position.

### 3.4. Pseudofactins

These are a class of lipopeptides produced by *Pseudomonas fluorescens.* There are of two major types—Pseudofactin I and Pseudofactin II. This class has got a huge therapeutic usage, especially Pseudofactin II, which has got antiadhesive properties [89]. Itsgreater emulsification activity and its stability have made it a compound of choice over other synthetic surfactants; therefore, it is considered potent in bioremediation. Structurally, Pseudofactins are cyclic octapeptides attached to palmitic acid [75]. 

### 3.5. Viscosins

Viscosins are also obtained from *Pseudomonas fluorescens* and have antibacterial and antifungal activity. The specific feature of viscosins is itshigh surface-activeness, which can inhibit the migration of cancer cells. In the case of *Pseudomonas*, viscosins increase the efficiency of plant roots and also have protective roles for germinating seedlings against plant pathogens [90]. 

### 3.6. Daptomycins

Daptomycins are a newer class of lipopeptide antibiotics approved by the US FDA and are effective against Gram-positive bacterial infections [91]. Structurally, daptomycins are a cyclic decanoyl lipid chain with 13 amino acid peptides. Theyexhibit broad-spectrum activity against an array of Gram-positive bacteria such as *Staphylococcus*, *Streptococcus*, *Pneumococcus*, *Clostridium*, and *Enterococcus*. The source microorganism for the production of daptomycin is actinobacterium *Streptomyces roseosporus* [91]. It inhibits the synthesis of lipoteichoic acid and disrupts bacterial membrane potential (depolarization) by the formation of pores which provides itsantimicrobial, antiparasitic, and immuno-suppressor properties. The functioning of daptomycin is calcium ion-dependent [91]. 

### 3.7. Poaeamides

Poaeamides are known fortheir diverse capability like swarming, biofilm formation, and regulation of attachment-detachment to plant roots, etc., apart from antifungal activity against plant pathogens like *Rhizoctonia solani* causing damping-off [92,93]. It is produced by *Pseudomonas* spp. [94] and has got potential pharma and biocontrol properties.

## 4. Biocontrol Potential of Lipopeptides

Almost all of the *Bacillus* species produce antimicrobial compounds known as lipopeptides and several strains of *B. subtilis* and *B. amyloliquefaciens* are reported to produce lipopeptides. Gong et al. [95] and Qian et al. [96] reported that the crude lipopeptides are stable to heat, pH, and showed high capability as biocontrol agents against various pathogens [97]. Mora et al. [60] observed that the antagonistic activity between plant-associated *Bacillus* and phyto-pathogens are related to the presence of cyclic lipopeptide genes. However, natural *Bacillus* strains play an important role in the production of different concentrations of each lipopeptide and thus are crucial for their interaction with plant as well as biofilm formation interaction with plants and the production of biofilms [85,98]. It has been reported that lipopeptides have shown potential antagonistic activity against disease causing bacteria and fungi *in vitro* and *in planta* conditions [99]. Cho et al. [100] reported that the *Bacillus pumilus* strain HY1 hasshown strong biocontrol activity against harmful aflatoxin producing fungi *A. flavus* and *A. parasiticus* suppress the fungal species *Aspergillus flavus* and *A. parasiticus*, which areproducers of potentially harmful aflatoxins. There are a few key mechanisms discussed in this section by which lipopeptides show antimicrobial activities (Table 2). 

### 4.1. Lipopeptides as Biosurfactants Distressing Membrane Integrity and Permeability 

Lipopoetide surfactants exhibited a unique pore and ion channels forming property thus may disturb the normal integrity and permeability of lipid bilayer of cell membrane [152]. This ability of disrupting the structural integrity of the biological membrane establishes their primary mode of antimicrobiotic action against bacteria, fungi, virus, mycoplasma, etc. [153]. Surfactins, which are one of the most potent biosurfactants, get inserted into the lipid bilayer after dimerization, chelate mono and divalent positively charged ions disturb the membrane permeability due to trans-membrane ion influxes and, finally, cause cell death as a result of cell disruption [153,154,155,156]. 

Iturins are strongly mycotoxic against a broad range of yeasts and fungal pathogens including *A. flavus* and *R. solaniin vitro*, but possess limited antibacterial and no activity against viruses [77,157,158,159]. Although iturins function through their membrane permeabilization properties, their mode of action is slightly different from surfactins [28]. Iturins cause osmotic disturbance by forming ion-conducting pores in the membrane without disrupting or solublizing them, unlike surfactins [160]. Fengicins are potent antifungal agents which act specifically against filamentous fungi, including phytopathogenic ones, *viz. Plasmodiaophora monoliforme, Fusarium moniliforme, Fusarium gramineareum*, and *Podosphera fusca* [161,162,163]. Their interaction with the lipid bilayer depends upon their concentration and they lead the alteration of membrane structural integrity and permeability [164].

Studies using scanning electron and optical microscopy revealed that *B. thuringiensis* CMB26 derived antibiotic lipopeptide affected the cell surface of plant pathogenic fungus, *Colletotrichum gloeosporioides*, *E. coli* O157, and *Pieris rapae crucivora* (cabbage white butterfly larvae) by acting on plasma membrane [128]. *Pseudomonas syringae* pv. *syringae* derived cyclic lipodepsipeptides, Syringopeptin, and Syringomycin also exhibited ion channel formation and lytic against plant and human cells due to their membrane-permeabilizing properties [165,166,167]. A fungal cyclohexadepsipeptides enniatin, derived from *Fusarium* sp. *Verticillium* and *Halosarpheia*, acts as an ionophore which first gets incorporated in the cell membrane and leads to the intracellular ion leakage and cation specific pores formation. This may establish the basis of the mechanism of actionof enniatin as a antimicrobial, anthelmintic, anticancer, and enzyme inhibiting agent [168].

Disruption of the integrity and permeability of biological membranes due to thepore and ion channels forming ability of lipopeptide surfactants has been established as the most important mode or mechanism of their action for explaining their applications as antibacterial, antifungal, antitumor, and hemolytic agents in the agriculture, biomedical, pharmaceutical, and therapeutic sectors. Moreover, the synergistic effect of various lipopeptides (mainly surfactins, iturins, and fengycins) showed antiadhesive activity, resulting in the reduction of colonization and stimulating biofilm dispersion of pathogenic bacteriadue to their amphiphilic surfactant like property [169]. The biosurfactant property of various lipopeptides is important in prohibiting biofilm formation. These biosurfactants are amphiphillic molecules that not only inhibit biofilm formation, but also dislodge the existing biofilm. Two *Pseudomonas fluorescens* lipopeptides putisovin I and II are reported to suppress the biofilm formation and breakdown of existing biofilm [170]. Abdallah et al. [171] reported the suppression of *Agrobacterium tumefaciens* biofilm by a mixture of lipopeptides produced by *Bacillus amyloliquifaciens.* The mixture maybe able to inhibit tumor formation upon pathogen adhesion on a tomato stem. In this experiment, the formation of new biofilm was also inhibited and the old ones dislodged. In agriculture, antiadhesive properties of various lipopeptides biosurfactants may also be used to reduce the attachment of phytopathogens on the plant surface and thus, their colonization efficiency, which is very crucial for the development of various plant diseases.

### 4.2. Lipopeptides as Siderophores

Iron is an essential nutrient that is required for normal functioning of important physiological processes including biological nitrogen fixation, transportation of oxygen, methane production, and DNA biosynthesis [172]. Microbes, including many bacteria and fungi, produce siderophores, a class of low molecular weight compounds with astrong tendency to form complexes with inorganic iron ions, thus making it biologically available to carry out cellular functions [173,174]. Several lipopeptides are also known to function as siderophores. Variochelins, a class of photoreactive lipopeptide siderophores, are mainly produced by specific genera of marine bacteria, *viz. Halomonas, Marinobacter, Ochrobactrum, Synechococcus*, and *Vibrio* [175,176,177,178]. Using a genome mining strategy, *Variovorax boronicumulans* BAM-48, a plant-associated terrestrial bacterium wasalso found to produce variochelins [151]. These siderophores support the growth of the producing organism by making important nutrients such as iron biologically available to them and also play a crucial role in determining the microbial community structure [179,180]. Some lipopeptide siderophores also act as chemical mediators for bacteria−algal interactions in the ocean [181]. These molecules have iron-binding α-hydroxycarboxylate ligand groups triggering a ligand-to-metal charge transfer reaction by absorbing photons in UV light, making iron available to surrounding microalgae, which, in turn, provides organic matter to the siderophore producing bacteria [181,182,183,184,185]. This mutualism has important ecological implications [186]. 

Moreover, *Herbaspirillum seropedicae* Z67 (class *Betaproteobacterium*), a N_2_ fixing and growth promoting endophyte, inhabiting many important crop has been reported to secrete a class of amphiphillic lipopeptidal siderophore called serobactins [187,188,189,190]. Serobactins employs a similar mechanism of increasing the bioavailability of inorganic iron common to many siderophores produced by aquatic bacteria [173]. The lipopeptide siderophores producing plant-associated bacteria can significantly influence the microbial community structure and thus, contribute to plant growth and health by modulating plant-microbe as well as plant-pathogen interactions.

### 4.3. Lipopeptides ISR-Inducer in Plants

Some beneficial bacteria indirectly protect plants from disease-causing microbes through the stimulation of inducible defense mechanisms which are systemic in nature and called ISR, which are effective in the management of several plant diseases [191]. Lipopeptides also showed antifungal activity and are involved in ISR activation and defense responses [116]. They are less toxic, biodegradable, and environmentally-friendly, with a broad range of target phytopathogens and, thus, have huge potential for plant diseases management. Many strains of *Bacillus* spp. strains are known to induce defense responses in plants, but knowledge about the molecular determinants of the *Bacillus* mediated ISR is very limited [36,192]. Ongena et al. [46] showed that surfactins and fengycins lipopeptides protect bean and tomato plants through ISR and, thus, represent a novel class of microbial-associated molecular patterns (MAMPs) which play an important role in the activation of the defense signalling pathway. They also revealed that surfactin and fengycin encoding genes when overexpressed in *Bacillus subtilis* strain 168 (poor producer) elevated the ISR potential of derivatives in tomato and bean plants. Moreover, increase in the activity of the main enzymes of the lipoxygenase pathway were observed in resistant plants when challenged with lipopeptide overproducers. They also hypothesized that surfactins and fengycins should have a distinct mechanism for ISR-induction in plants. 

**Figure 3:**
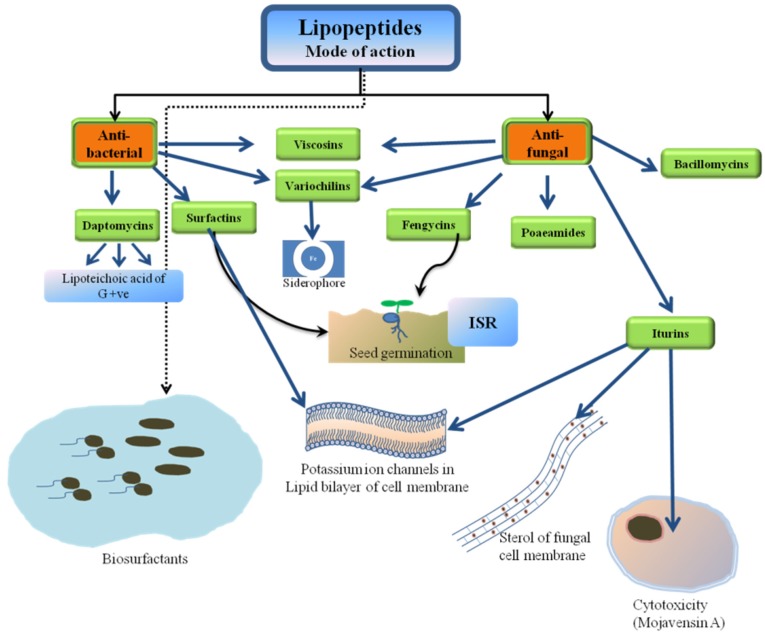
The possible mechanisms of lipopeptide mediated plant disease management.

Surfactin and fengycin caused ISR induction in the plants against phytopathogenic fungi, but showed varying responses in different plant cell types [28]. Now, it is well established that surfactin is crucial for ISR induction, colonization of root and biofilm formation and extracellular matrix formation in *B. subtilis* [193]. The binding of surfactin molecules to the plant cell membrane is mainly responsible for ISR-induction [194]. Enhanced resistance has been reported againstgrey leaf spot disease caused by *Magnaporthe oryzae* due to H_2_O_2_-mediated defense responses sensitized by ISR responses elicited by semi-purified surfactin lipopeptides in perennial ryegrass [195]. The *Pseudomonas*-derived cyclic lipopeptide orfamide was shown to act as an ISR elicitor, triggering early defense events and inducing expression of defence related genes without causing cell death in rice against the brown spot disease fungus *Cochliobolus miyabeanus* [196]. Endophytic bacteria showed antifungal activity, which played an important role indefending plants against invading fungal pathogens. Endophytic *Bacillus* sp. has been utilized in protecting maize and horse-bean fungal pathogens. Gond et al. [57] reported that through lipopeptide production, the maize associated symbiotic bacteria directly inhibits the potential pathogens and also induce host defense gene activation against fungal pathogens. The possible mechanisms of lipopeptide mediated plant disease management are summarized in Figure 3.

## 5. Future prospects of Microbial Lipopeptides in Plant Disease Management

Cropdestruction by existing and emerging phytopathogens is an important issue of contemporary agriculture that needs to be addressed properly to optimize gains from this age-old enterprise. As the population continues to build up, losses due to plant diseases are emerging as a threat to global food security. Plant diseases affect agricultural produce not only quantitatively, but also qualitatively, hence affecting the gains from marketable produce at two distinct levels. Not only this, but food safety is also a concern that is related to microbial infestation of agricultural produce [197]. Many of the currently available antimicrobial products that areused in agriculture are highly toxic and non-biodegradable and, thus, cause extended environmental pollution. Moreover, increasing resistance to existing antimicrobial agents among the phytopathogens is also a matter of great concern for the future of agriculture [84,198]. On the other hand, extensive use of agrochemicals has disturbed the ecological balance by creating non-target toxicity, development of resistance among pathogens, contamination of reservoirs and groundwater, obvious health risks to humans and other living beings, and increases in the cost of cultivation. In such a situation, the challenge before microbiologists and plant pathologists in the future is to control the stronger pathosystems using environmentally-friendly alternatives. This would require exploring non-conventional and newer approaches to combat a variety of crop diseases [46]. Plant resistance has been exploited for along timeand in response, a large number of resistant strains of plant pathogens have been reported. The current scenario of antimicrobial agents involvesthe problem of toxic effects on non-target organisms, including human consumers and the environment, and this toxicity, coupled with low biodegradability, has made this solution poor [199] enough to search for alternatives. 

Microbial agents for biological control are being explored extensively in different crops against an array of pathogens as they exhibit a wider range of antimicrobial activities. Some microbial agents directly act upon the pathogens by millions of antimicrobial compounds, nutrient quenching, competition for space, etc. Whereas, others interact with plants with long-distance signaling by eliciting defense responses using microbe-associated molecular patterns and their compounds or priming plants without making any direct interaction to the pathogen in question. These beneficial microbes modulate the growth condition of the surrounding region so that the pathogen could not thrive and develop in numbers to cause economically significant damage to plants. 

Antagonistic microbes control the pathogens *via* hyperparasitism and antibiosis in direct interaction. This involves multiple mechanisms and compounds generated through highly regulated cascades. To use these antimicrobial metabolites for the control of pathogens on the field requires a stringent and exhaustive registration process to avoid any non-target effects on the other constituents of the environment. In this aspect, currently, risks associated with antagonistic microbial metabolites are often assessed similar to that of single-molecule fungicides. Since the nature of the compound and mode of actions are different, thisrequires a re-thinking of data requirements for the registration of microbial agents as biopecticides. Endless research data indicated the enormous antimicrobial capabilities of the genus *Bacillus, Pseudomonas*, and *Trichoderma.* These three genera are considered as factories for the production of biologically active molecules which are potent growth inhibitors of phytopathogens. Lipopeptides, a subclass of antimicrobial peptides, arenow emerging as an attractive alternative for the development of new peptide-based biopesticides [84,200]. The usefulness of lipopeptides is being evaluated as a potent versatile weapon for the control of a wide range of phytopathogens. The three broad families of *Bacillus* lipopeptides, surfactins, iturins, and fengycins, along with other new groups of antimicrobial lipopetides are being explored for their effectiveness against a wide range of phytopathogens including bacteria, fungi, and viruses [84]. Shafi et al. [201] advocated that the *Bacillus* species has numbers of antagonizing attributes against plant pathogens including the production of lipopeptides, enzymes, and plant growth promotion. Hazarika et al. [202] studied the role of lipopeptides produced by leaf endophytes against 10fungal species.

Apart from bacterial lipopetides, fungi-derived lipopeptide antibiotics are also gaining importance. These lipopetides have four categories: cyclic depsipeptides, peptaibiotics, non-depsipeptide cyclic lipopeptides, and non-peptaibiotic linear lipopeptides [203,204]. These compounds have greater future significance due to their varied bioactivity and diversity. The major fungal genera producing lipopetides are *Acremonium*, *Aspergillus*, *Alternaria*, *Metarhizium, Beauveria*, *Fusarium, Penicillium*, etc. They exhibit cytotoxic, antimicrobial, antiviral, insecticidal, antitumoral, and enzyme-inhibitory activities which could be potential weapon against plant pathogens of economic importance [203]. Fungi derived antimicrobial lipopetides like peptaibols, lipoaminopeptides, lipopeptaibols, echinocandins, aspochracins, etc. could be tested against new and emerging pathosystems for preparing biofungicides of tomorrow. 

This could open an entire new arena of biopesticides since the whole of society is concerned about green chemicals. These biodegradable biosurfactants could be environmentally-friendly with low toxicity andalternatives to highly toxic synthetic chemical pesticides [205]. The implications of overwintering and the resting stages of pathogens could also be worked out to reduce the inoculum loads from croplands. The microbial strain may be engineered for the novel structure of surfactin production [206]. Further, advancement in the bioinformatics assisted molecular and dynamics simulation studies can also help to decipher the molecular and biochemical mechanisms. Moreover, several reports have already been initiated on the structure prediction and their possible interaction with different cellular protein/enzyme targets [207,208,209,210]. Cob-Calan et al. [211] demonstrated the interactions of the cyclic lipopeptides iturin A, fengycin, and surfactin with β-tubulin using molecular docking and molecular dynamics simulation. A comparative study had shown that iturin A and fengycin had higher binding affinity as compared to surfactin for the catalytic site of β-tubulin [210,211]. With the help of advancements in genetic engineering and synthetic biology, improved lipopeptide production with higher efficiency could be developed, targeting important pathosystems. The work on reducing the cost of industrial production is also an area to be explored with greater efficiency. With all these initiatives, the lipopeptides of microbial origin could form a useful and wider base to combat the plant pathogens of today and tomorrow. 

## Figures and Tables

**Table 1:** List of various lipopeptides encoding genes and their respective primer set for PCR amplification.

S.No.	Lipopeptide Class	Gene	Primer Name	Primer Sequences (5′-3′)	Reference
**1.**	**Surfactins**				
(a)	Surfactin	*sf*P	SFP-F1 SFP-R1	ATGAAGATTTACGGAATTTA TTATAAAAGCTCTTCGTACG	[58]
(b)	Surfactin	*Srf*c	Sur3f Sur3r	ACAGTATGGAGGCATGGTC TTCCGCCACTTTTTCAGTTT	[57]
(c)	Surfactin	*Srf*A-A	srfAA-Fw srfAA-Rv	AAAGGATCCAGCCGAAGGGTG TCATGGT AAAAAGCTTGTTTTTCTCAAAGAACCAGCG	[59]
(d)	Surfactin	*srf*AA (Surfactin synthetase subunit 1)	SRFAF SRFAR	TCGGGACAGGAAGACATCAT CCACTCAAACGGATAATCCTGA	[60]
(e)	Surfactin	*srf*A	F3726 R3879	GAAGTCTTCAGCGGCGAACTG GGGTGGCTCCGTTTTTCTCG	[56]
(f)	Surfactin	*srf*DB	SUR3F SUR3R	ACAGTATGGAGGCATGGTC TTCCGCCACTTTTTCAGTTT	[61]
**2.**	**Iturins**				
(a)	Iturin A	*Itu*D	ItuD1f ItuD1r	GATGCGATCTCCTTGGATGT ATCGTCATGTGCTGCTTGAG	[57]
(b)	Iturin A	*Itu*-C	ituC-Fw ituC-Rv	AAAGGATCCAAGCGTGCCTTTTACGGGAAA AAAAAGCTT AATGACGCCAGCTTTCTCTT	[59]
(c)	Iturin	*itu*C (Iturin A synthetase C)	ITUCF ITUCR	GGCTGCTGCAGATGCTTTAT TCGCAGATAATCGCAGTGAG	[60]
**3.**	**Fengycins**				
(a)	Fengycin	*Fen*D	FenD1f FenD1d	TTTGGCAGCAGGAGAAGTTT GCTGTCCGTTCTGCTTTTTC	[62]
(b)	Fengycin	*fen*D (Fengycin synthetase)	FENDF FENDR	GGCCCGTTCTCTAAATCCAT GTCATGCTGACGAGAGCAAA	[60]
(c)	Fengycin	*Fen*E	FenEF FenER	GTTTCATGGCGGCGAGCACG GATTCGCGGGAAGCGGATTGAGC	[62]
(d)	Fengycin	*Fen*	Af2-F Tf1-R	GAATAYMTCGGMCGTMTKGA GCTTTWADKGAATSBCCGCC	[63]
**4.**	**Bacillomycins**				
(a)	Bacillomycin	*bmy*B (Bacillomycin L synthetase B)	BMYBF BMYBR	GAATCCCGTTGTTCTCCAAA GCGGGTATTGAATGCTTGTT	[60]
(b)	Bacillomycin D	*Bam*C	Bacc1f Bacc1r	GAAGGACACGGAGAGAGTC CGCTGATGACTGTTCATGCT	[57]
(c)	Bacillomycin D	*bam* D	ITUD-F1 ITUD-R1	TTGAAYGTCAGYGCSCCTTT TGCGMAAATAATGGSGTCGT	[64]
**5.**	**Bacilysin**				
(a)	Bacilysin	*bacAB*	BACD-F1 BAMD-R1	AAAAACAGTATTGGTYATCGCTGA CCATGATGCCTTCKATRCTGAT	[65]
(b)	Bacilysin	*bacAB*	BACAB-F1 BACAB-R1	CTTCTCCAAGGGGTGAACAG TGTAGGTTTCACCGGCTTTC	
**6.**	**Ericin**				
	Ericin	*eriB*	*eriBF* *eriBR*	GAWKNACWCCWTWTGG CCRCCATATCSWTMTRYYTC	[66]
**7.**	**Mersacidin**				
(a)	Mersacidin	*mrs*A	MRSA-F1 MRSA-R1	GGGTATATGCGGTATAAACTTATG GTTTCCCCAATGATTTACCCTC	[67]
(b)	Mersacidin	*mrs*M	MRSM-F1 MRSM-R1	AAATGACCCGGCATATGTTC TGCTGACTAACTGGAATTGGAA	
**8.**	**Mycosubtilin**				
(a)	Mycosubtilin	*fen*F	ITUD-F1 ITUD-R1	TTGAAYGTCAGYGCSCCTTT TGCGMAAATAATGGSGTCGT	[68]
(b)	Mycosubtilin	*myc*C	MYCC-F1 MYCC-R1	AATCAATTGGCACGAACCTT ATCGCCCGTTTTGTACATTC	
**9.**	**Zwittermicins**				
(a)	Zwittermicin A	*Zwit*	ZWITF2 ZWITR1	TTGGGAGAATATACAGCTCT GACCTTTTGAAATGGGCGTA	[61]
**10.**	**Kurstakins**				
(a)	Kurstakins	*Kur*	Aks-F Tks-R	TCHACWGGRAATCCAAAGGG CCACCDKTCAAAKAARKWATC	[69]

Where H denotes A or C or T, W-A or T, R-A or G, D- A or G or T, K- G or T, Y-C or T.

**Table 2:** Different lipopeptides, their source microorganisms, and the nature of antimicrobial activity.

S. No.	Source Organism	Lipopeptide Class/Type	Activity/Action	References (Not to Be Attended)
1.	*Actinoplanes friuliensis*	Friulimicin	Broad range of multi-resistant Gram-positive bacteria	[101]
2.	*Arthrobacter* spp. MIS38	Arthrofactin	Bio-surfactant	[102]
3.	*Bacillus subtilis*	Iturin A, Bacillomycin, Fengycin, Bacillomycin	Antifungal	[57]
4.	*B. subtilis* HC8	Surfactin, Fengycin A, Fengycin B, Iturin A,	Antifungal	[52]
5.	*B. subtilis* K1	Surfactin, Iturin, Fengycin A and B, Fengycin A2 and B2	Antifungal	[103]
6.	*B. subtilis* GA1	Iturins, Fengycins, Surfactins	Antifungal	[104]
7.	*B. subtilis* M4	Fengycin A and B	Antifungal	[30]
8.	*B. subtilis* B-FS01	Fengycin	Antifungal	[105]
9.	*B. subtilis* and *B. amyloliquefaciens*	Fengycin, Iturins, Surfactins, Bacillomycin	Antibaterial	[60]
10.	*B. subtilis* SPB1	Surfactin, Fengycin, Iturins	Antifungal	[106]
11.	*B. subtilis* EBS05	Surfactin A	Antifungal	[107]
12.	*B. subtilis* B49	Fengycin, Bacillomycin D	Antifungal	[61]
13.	*B. subtillis* ATCC 13952	Fengycin	Antifungal	[61]
14.	*B. subtillis* DF-HO8	Fengycin	Antifungal	[61]
15.	*B. subtilis* CMB32	Iturin A, Fengycin, Surfactin A	Antifungal	[108]
16.	*B. subtilis* (Marine)	Surfactins and Fengycins	Delayed Germination	[109]
17.	*B. subtilis* B1	Iturin C, Surfactin, Fengycin A and B, Bacillomycin D, Bacilysin, Mycobacillin	Antifungal	[110]
18.	*B. subtilis* JA	Surfactin, Iturin, and Fengycin	Antifungal	[111]
19.	*B. subtilis* 9407	Fengycin	Antifungal	[112]
20.	*B. subtilis* HC8	Fengycin A and Fengycin B	Antifungal	[52]
21.	*B. subtilis* S499	Surfactin, Fengycin A, and Fengycin B	Antifungal	[30]
22.	*B. subtilis* fmbj	Fengycin A and Fengycin B	Antifungal	[113]
23.	*B. subtilis* EPCO16	Iturin A, Surfactin, Zwittwermicin A, Bacillomycin D	Antifungal	[114]
24.	*B. subtilis* 6051	Surfactin	Antibacterial activity against *P. Syringae*	[79]
25.	*B. subtilis* M4	Iturin/Fengycin	Antifungal activity against *Pythium ultimum* causing Damping-off disease of Beans	[30]
26.	*B. subtilis* M4	Fengycin	Antifungal activity against *Botrytis cinerea* causing Gray mold disease of Apples	[30]
27.	*B. subtilis*	Iturin/Fengycin	Antifungal activity against *podosphaera fusca* causing Powdery mildew of Cucurbits	[97]
28.	*B. subtilis* JA; JA026	Fengycin	Antifungal activity against *Gibberella zeae* (anamorph of *Fusarium graminearum*) Fusarium causing head blight (FHB) in Wheat and Barley and Ear rot in Corn	[115]
29.	*B. subtilis* B-FS01	Fengycin	Antifungal activity against *Fusarium moniliforme* causing Seedlingblight, Stalk rot, and Ear rot	[105]
30.	*B. subtilis* BBG127 and BBG131	Cyclic lipopeptides	Antifungal activity against *Botrytis cinerea* 630 causing Necrosis of Grapevines	[116]
31.	*B. subtilis* 9407	Fengycin	Antifungal activity against *Botryosphaeria dothidea* causing Apple ring rot	[112]
32.	*B. subtilis* GA1	Iturin, Fengycin, Surfactin	Antifungal activity against *Botrytis cinerea* causing Grey mould disease of Apples	[104]
33.	*B. amyloliquefaciens* ES-2	Fengycin, Surfactin	Antibacterial/anti-fungal	[53]
34.	*B. amyloliquefaciens* TF28	Iturin A	Antifungal	[54]
35.	*B. amyloliquefaciens* ARP23 and MEP218	Surfactin C15, Fengycins A, Iturin A	Antifungal activity against *Sclerotinia sclerotiorum*	[59]
36.	*B. amylolequifaciens* S499	Fengycin, Iturins, Surfactin	ISR	[117]
37.	*B. amyloliquefaciens* 32a	Surfactin, Iturin A, Bacillomycin D, Fengycin	Antimicrobial	[118]
38.	*B. amyloliquefaciens* CC09	Fengycin, Iturin, Surfactin, Bacillomycin	Antifungal	[18]
39.	*B. amyloliquefaciens* BO7	Surfactin	Antifungal	[119]
40.	*B. amyloliquefaciens* subsp. *plantarum* SV65	Fengycin	Antifungal activity	[120]
41.	*B. amyloliquefaciens* MEP218	Iturin, Fengycin, Surfactin	Antibacterial, Antifungal	[121]
42.	*B. amyloliquefaciens* KPS46	Surfactin	Antibacterial activity against *Xanthomonas axonopodis* pv. *glycines*	[122]
43.	*B. amylolequifaciens*	Lipopeptides, Surfactin, Iturin, Fengycin	Antiviral ActivityagainstRhizomania, an important disease of Sugarbeet	[117]
44.	*B. mojavensis* RRC101	Leu7-Surfactin	Antifungal	[123]
45.	*B. mojavensis* A21	Surfactin, Fengycin, Pumalicidin	Antimicrobial, Antifungal	[124]
46.	*B. mojavensis* RRC101	Surfactin, Fengycin	Antifungal	[125]
47.	*B. licheniformis*	Lichenysin	Bio-surfactant	[124]
48.	*B. licheniformis*	Surfactins, Lichenysins	Bio-surfactant	[126]
49.	*B. pumilus* HY1	Iturins	Antifungal	[100]
50.	*B. pumilus* (Marine)	Pumilacidin	Antibacterial activityagainst *Staphylococcus aureus*	[127]
51.	*B. thuringiensis* CMB26	Fengycins	Fungicidal, Bactericidal, andInsecticidal activity	[128]
52.	*B. thuringiensis kurstaki* HD-1	Kurstakins	Antifungal activity against *Stachybotrys charatum*	[129]
53.	*B. thuringiensis kurstaki*	Kurstakin	Antifungal activity	[130]
54.	*B. vallismortis* R2	Surfactins, Iturins, Fengycins	Antifungal activity against *Alternaria alternate* causing Black point disease of Wheat	[131]
55.	*B. cereus* DFE4	Surfactin, Iturin A, Bacillomycin D	Antifungal	[132]
56.	*B. methyltrophicus* TEB1	Iturin A, Fengycin, Surfactin	Antifungal	[133]
57.	*B. methylotrophicus* HC51	Iturin A, Fengycin	Antifungal	[134]
58.	*Bacillus* sp.C3	Iturin A, Surfactin, Subtilosin, Subtilin	Antifungal	[135]
59.	*Bacillus* sp.BmB9	Surfactin, Iturin, Fengycin	Antifungal, Antibacterial	[136]
60.	*Bacillus* sp. FJAT-14262	Surfactin	Antifungal	[137]
61.	*Bacillus* sp. CY22	Iturin	Antifungal	[138]
62.	*Bacillus* sp. NH-100	Surfactin A	Antifungal	[139]
63.	*Bacillus* sp.	Iturin A, Surfactin, Zwittermicin A, Bacillomycin D	Antifungal activity against *Fusarium oxysporum* f.sp. *lycopersici* causing Wilt in Tomato	[114]
64.	*Bacillus* sp. (Marine)	Mixirins A, B, and C	Cytotoxic	[77]
65.	*Chromobacterium* sp. C61	Chromobactomycin	Antifungal activity against *Magnoporthe grisea* causing Rice Blast	[140]
66.	*Fusarium* sp. YG-45	Fusaristatins A and B	Antimicrobial	[141]
67.	*Fusarium decemcellulare* LG53	Fusaristatin A	Mildantimicrobial	[142]
68.	*Geitlerinema* sp.(Marine cyanobacterium)	Mitsoamide	Cytotoxic activities	[77]
69.	*Herbaspirillum seropedicae* Z67	Serobactins A, B, and C	As aniron source	[143]
70.	*Pseudomonas fluorescens* 96.578	Tensin	Antifungal activity against *Rhizoctonia solani* causing Sugarbeet seed infection	[144]
71.	*P. fluorescens* BD5	Pseudofactin II	Anti-adhesive activity	[89]
72.	*P. fluorescens* SS101	Massetolide A	Systemic resistance (Late Blight)	[45]
73.	*Pseudomonas poae* RE*1-1-14	Poaeamide	Antifungal activity against *Rhizoctonia solani* causing Damping off and Rootrot in Sugarbeet	[93]
74.	*Pseudomonas* sp. UCMA 17,988 (Isolated from Bovine raw milk)	Milkisin	Antimicrobial activity against *Listeria monocytogenes, Staphylococcus aureus*, and *Salmonella enteric*	[145]
75.	*Paenibacillus polymyxa* M-1	Polymyxin, Fusaricidin	Suppress phytopathogenic *Erwinia* spp.	[146]
76.	*Paenibacillus* sp. IIRAC-30	Surfactin	Antifungal	[147]
77.	*Scopulariopsis brevicaulis* (Marine sponge-derived)	Scopularides A and B	Cytotoxic activities	[77]
78.	*Streptomyces canus*	Amphomycins	Inhibit bacterial cell wall synthesis	[148,149]
79.	*Streptomyces viridochromogenes*	Laspartomycins	Antibacterial, Antiherpes activity	[150]
80.	*Variovorax boronicumulanss* BAM-48	Variochelins A and B	Siderophore production	[151]
